# Integrative Machine Learning and Experimental Validation Identify FIS1 as a Candidate Biomarker Linked to Mitochondrial Dynamics in Pulmonary Hypertension

**DOI:** 10.3390/cells15030301

**Published:** 2026-02-05

**Authors:** Yu Zhang, Qing Dai, Lijun Gong, Runxiu Zheng, Wei Huang, Feiying Wang, Rong Yuan, Lan Song, Aiguo Dai

**Affiliations:** 1 School of Integrated Chinese and Western Medicine, Hunan University of Chinese Medicine, Changsha 410208, China; yuzhang@stu.hnucm.edu.cn (Y.Z.);; 2 Hunan Provincial Key Laboratory of Vascular Biology and Translational Medicine, Changsha 410208, China; 3 Department of Respiratory Medicine, School of Medicine, Changsha 410021, China

**Keywords:** pulmonary hypertension, mitochondrial dynamics, FIS1, machine learning, ferroptosis

## Abstract

**Highlights:**

**What are the main findings?**

- Integrative transcriptomic analyses with machine learning prioritized FIS1 as a candidate molecular signal associated with mitochondrial dynamics alterations in pulmonary hypertension.
- In hypoxia-related PH models, increased FIS1 expression was accompanied by enhanced mitochondrial fission, increased oxidative stress, and altered pulmonary artery smooth muscle cell behavior.

**What are the implications of the main findings?**

- The findings support a mechanistic connection between disrupted mitochondrial dynamics and ferroptosis-associated stress features in hypoxia-related PH models.
- This study provides testable hypotheses and molecular leads for future validation of mitochondria–ferroptosis crosstalk and potential intervention strategies in pulmonary hypertension.

**Abstract:**

Pulmonary hypertension (PH) is characterized by progressive pulmonary vascular remodeling and a paucity of effective therapeutic interventions. Although dysregulated mitochondrial dynamics are implicated in this remodeling process, the key regulatory molecules and downstream mechanisms remain incompletely defined. This study aimed to systematically characterize molecular alterations associated with mitochondrial dynamics in PH and to explore the functional relevance and potential mechanisms of prioritized candidate genes. We integrated transcriptomic datasets from PH models with MitoCarta annotations to identify mitochondria-related differentially expressed genes. Candidate genes were prioritized using WGCNA and three machine-learning algorithms (LASSO, SVM-RFE, and random forest). These candidates were then experimentally evaluated in a hypoxia-induced PH mouse model and hypoxia-stimulated mouse pulmonary artery smooth muscle cells (mPASMCs) using qRT–PCR, Western blotting, immunohistochemistry, and transmission electron microscopy. Functional assays and assessments of mitochondrial injury were performed to investigate pathogenic relevance. Our analysis identified four key genes, with FIS1 showing high ROC/AUC-based discriminatory performance in both the training dataset and the independent replication dataset. Hypoxia was associated with increased FIS1 expression, mitochondrial fragmentation, loss of mitochondrial membrane potential, and ROS accumulation. We further observed that FIS1 knockdown suppressed mPASMC proliferation and migration, alleviated mitochondrial injury, and attenuated ferroptosis-associated alterations, accompanied by reduced lipid peroxidation, decreased Fe^2+^ accumulation, and partial normalization of ferroptosis-related marker proteins. Taken together, these findings suggest that FIS1 may contribute to PH pathogenesis through mitochondrial fission and ferroptosis-associated stress, potentially promoting aberrant PASMC phenotypes and pulmonary vascular remodeling. This work provides a mechanistic rationale and molecular leads that may inform molecular stratification and mechanistically informed therapeutic exploration targeting mitochondrial pathways in PH.

## 1. Introduction

Pulmonary hypertension (PH) is a progressive cardiopulmonary disorder characterized by sustained elevations in pulmonary arterial pressure and pulmonary vascular resistance, ultimately leading to right ventricular failure and mortality [1]. Epidemiological evidence indicates that PH affects approximately 1% of the global adult population and remains associated with poor clinical outcomes [2]. Although approved agents such as bosentan and sildenafil are available, treatment responses are heterogeneous and adverse effects remain common, underscoring the need to identify novel targets and develop more effective therapeutic strategies [3,4].

Pulmonary vascular remodeling (PVR) is a pathological hallmark of PH, featuring distal arterial wall thickening and luminal narrowing driven by endothelial dysfunction, smooth muscle cell remodeling, and inflammation [5]. In advanced disease, lesions may include plexiform changes and in situ thrombosis [5]. These alterations progressively narrow and occlude pulmonary arterioles, thereby increasing pulmonary vascular resistance and driving disease progression. However, the molecular programs governing PVR are not fully elucidated, which hampers the development of robust diagnostic stratification and targeted interventions. In parallel, a range of clinical and molecular biomarkers have been explored for disease assessment and risk evaluation in PH, including cardiac stress markers, functional and hemodynamic indices, and molecular signals related to endothelial dysfunction and inflammation [6]. Despite these advances, there remains a need for mechanistically grounded molecular candidates that better link disease biology with phenotyping and therapeutic exploration [7].

Mitochondria are central to cellular energy production and redox control and depend on coordinated quality-control programs, including fusion, fission, biogenesis, and mitophagy, to preserve mitochondrial structure and function. Disruption of mitochondrial dynamics, particularly a shift toward excessive fission or reduced fusion, can drive mitochondrial fragmentation and oxidative stress and has been implicated in a range of chronic disorders, including chronic respiratory diseases [8,9,10]. Emerging evidence further suggests that dysregulated mitochondrial dynamics may heighten cellular susceptibility to ferroptosis-related injury [11]. Ferroptosis is a regulated cell death modality characterized by iron-dependent lipid peroxidation, and mitochondrial dysfunction and redox imbalance can modulate ferroptosis-associated stress responses [12,13]. Together, these observations support the rationale that mitochondrial dynamic imbalance may contribute to vascular injury and remodeling in pulmonary hypertension.

Fission 1 (FIS1) is an outer mitochondrial membrane protein that helps organize the mitochondrial fission machinery and has been implicated in Drp1-dependent fission processes [14]. Although aberrant mitochondrial fission has been reported in experimental PH, the specific fission regulators in PASMCs that exhibit both candidate biomarker potential and mechanistic relevance to PVR remain incompletely defined [15]. Here, we aimed to identify genes related to mitochondrial dynamics with diagnostic and mechanistic relevance to pulmonary vascular remodeling in pulmonary hypertension, and we identified FIS1 as a candidate gene upregulated in pulmonary hypertension that is linked to mitochondrial injury in experimental models.

## 2. Materials and Methods

### 2.1. Data Collection and Processing

PH-related transcriptomic datasets were retrieved from the NCBI Gene Expression Omnibus (GEO) using the terms “pulmonary hypertension”, “hypoxic pulmonary hypertension”, and “idiopathic pulmonary hypertension (IPH)”. Eligible studies were required to provide expression matrices with sample annotations (microarray or high-throughput sequencing; *Homo sapiens* or *Mus musculus*). Three datasets were included: GSE254617, GSE15197, and GSE117261.

GSE254617 (GPL21290) contains human explanted lung tissue samples; according to GEO annotations and the series description, 96 PH cases and 52 healthy donor lungs were retained as controls (total *n* = 148). GSE254617 was used as the training datasets, and GSE15197 (microarray; platform per GEO annotation) was used as an independent replication (validation) datasets. Where applicable, platform-specific probe identifiers were mapped to official gene symbols using the corresponding annotation files; when multiple probes mapped to the same gene, expression values were aggregated (median). Processed matrices provided by GEO were used when available; otherwise, expression values were transformed as appropriate for downstream analyses. ROC curves were generated and AUC values were calculated separately in GSE254617 and GSE15197 to assess ROC/AUC-based discriminatory performance of the selected candidate genes.

For integrative discovery analyses, GSE254617 and GSE15197 were aligned by shared gene symbols after harmonization, and batch effects attributable to dataset heterogeneity were removed using ComBat (sva package, 3.44.0) with dataset specified as the batch variable. The resulting batch-corrected matrix was used for WGCNA and subsequent machine learning based feature prioritization.

GSE117261 (single-cell RNA-seq; GPL20301 and GPL21103) contains single-cell transcriptomes from human pulmonary arteries, including healthy donors and PH samples. This dataset was leveraged to characterize cell-type resolved expression patterns of candidate genes; where appropriate for visualization and donor-level comparisons, single-cell profiles were aggregated to a donor-level pseudo-bulk matrix. We further checked the consistency of group labels and species designation prior to differential and integrative analyses.

### 2.2. Identification of MitoDEGs and Functional Enrichment Analysis

DEGs between PH and healthy controls (HC) were identified using DESeq2 (R package), with significance defined as |log2FoldChange| > 0.5 and adjusted *p*-value < 0.05. High-confidence human mitochondrial genes were retrieved from MitoCarta 3.0 (http://www.broadinstitute.org/mitocarta, accessed on 1 October 2025). Intersecting the DEG set with the MitoCarta 3.0 compendium yielded mitochondria-related DEGs (MitoDEGs) in PH. Up- and down-regulated MitoDEGs were analyzed separately for functional enrichment using the clusterProfiler (R package) and org.Hs.eg.db (R package), assessing Gene Ontology (GO) terms and Kyoto Encyclopedia of Genes and Genomes (KEGG) pathways. Enrichment results with adjusted *p*-value < 0.05 were considered statistically significant.

### 2.3. Weighted Gene Co-Expression Network Analysis

WGCNA was performed to delineate modules of co-expressed genes and relate them to clinical traits, following the framework of Langfelder and Horvath [16]. Samples were first hierarchically clustered to identify and remove outliers, thereby improving network stability. A signed, weighted adjacency matrix was constructed using a soft-thresholding power (β) selected according to the scale-free topology criterion, thereby emphasizing strong correlations while down-weighting weak ones. Topological overlap based hierarchical clustering was applied to the resulting network, and gene modules were detected using the dynamic tree-cut algorithm. For each module, the module eigengene (ME) was calculated and correlated with the clinical phenotype of PH. Module membership (MM; correlation between a gene and the ME) and gene significance (GS; correlation between a gene and the external trait) were computed to quantify the contribution of individual genes. Modules exhibiting the strongest trait associations and containing genes with MM > 0.8 and |GS| > 0.2 were designated as biologically relevant. Within these modules, hub genes were defined by high intramodular connectivity together with high MM, reflecting central roles in the co-expression network.

### 2.4. Identification of Hub Genes Using Machine-Learning Approaches

To further prioritize candidate classifier features pivotal to PH, we implemented support vector machine (SVM) modeling on the module genes most associated with the trait [17,18]. Feature selection was refined via SVM–recursive feature elimination (SVM-RFE), which iteratively removes the least informative predictors to maximize classification margin and improve generalization on high-dimensional data. Model hyperparameters were tuned by internal cross-validation, and the most parsimonious feature set yielding optimal performance was retained. Genes consistently selected by SVM-RFE and exhibiting high intramodular connectivity were designated as hub genes for downstream analyses.

### 2.5. Predictive Performance of Biomarkers in Pulmonary Hypertension

Receiver operating characteristic (ROC) curves were generated in R using the pROC package to evaluate the discriminatory performance of candidate biomarkers, and the area under the curve (AUC) was calculated accordingly. ROC/AUC was computed separately in the training dataset (GSE254617) and in the independent external replication dataset (GSE15197). Where applicable, 95% confidence intervals for AUC were estimated using DeLong’s method.

### 2.6. Immune Cell Infiltration and the Association Between Hub MitoDEGs and Differential Immune Cells in PH

To analyze leukocyte composition from bulk transcriptomes, we employed the CIBERSORT computational deconvolution algorithm, which calculates the relative abundance of immune cell types based on gene-expression signatures. In the newly assembled datasets, CIBERSORT was utilized to quantify the proportions of 22 distinct immune cell populations, followed by comparative analyses of their distributions between PH cases and healthy controls. Group differences were evaluated using appropriate statistical tests, with *p* < 0.05 considered statistically significant unless otherwise specified.

### 2.7. Animal Experiments

Seven-week-old male C57BL/6 mice were supplied by Hunan SJA Laboratory Animal Co., Ltd. (Changsha, China). The study utilized C57BL/6 mice individually housed under SPF-grade conditions at Hunan University of Traditional Chinese Medicine, with ad libitum access to sterilized food and water. The experimental protocols were reviewed and approved by the Institutional Animal Care and Use Committee on 16 October 2024 (ethical code HNUCM21-2510-15), confirming adherence to established ethical guidelines for animal research.

#### 2.7.1. Hypoxic Pulmonary Hypertension Mouse Model

Following the established protocol [19], we induced hypoxia-induced pulmonary hypertension (HPH) in C57BL/6 mice using intermittent normobaric hypoxia. The oxygen concentration in the chamber was precisely maintained at 10.0 ± 0.5% via dynamic nitrogen infusion. Chamber CO_2_ was absorbed using soda lime, and humidity was controlled with anhydrous calcium chloride. Animals were exposed to hypoxia for 8 h/day for 28 consecutive days. During hypoxic conditioning, mice were monitored daily for general health and behavior (activity, grooming, posture, and food/water intake), and body weight was recorded at regular intervals. No animals required early removal due to welfare concerns.

#### 2.7.2. Echocardiographic Assessment

Echocardiographic assessment was conducted using the VINNO6 ultrasound Doppler imaging system (Vinno 6, Vinno, Suzhou, China) via transthoracic echocardiography under light anesthesia, as previously described [20]. Briefly, mice were anesthetized with isoflurane (3.0% for induction and 1.5% for maintenance; Reverd Life Sciences, Shenzhen, China) and placed in the supine position on a heated pad to maintain body temperature. The thoracic area was depilated using depilatory cream to enhance acoustic coupling and facilitate image acquisition.

Pulmonary artery acceleration time (PAT) and pulmonary artery ejection time (PET) were measured by pulsed-wave Doppler in the parasternal long-axis view. Tricuspid annular plane systolic excursion (TAPSE) was assessed using M-mode imaging in the apical four-chamber view. All measurements were obtained at comparable heart rates to minimize physiological variability between groups.

#### 2.7.3. Hemodynamic Measurements and Right Ventricular Hypertrophy Index Assessment

Right ventricular systolic pressure (RVSP) was measured in mice by right heart catheterization via the external jugular vein, as previously described [21]. Briefly, a 1.0 F pressure-volume catheter (Millar Instruments, Houston, TX, USA) was inserted into the right ventricle via the right jugular vein to measure RVSP, as previously described. Prior to catheterization, the BIOPAC MP160 multichannel physiological recording system (BIOPAC Systems, Goleta, CA, USA) was calibrated [22,23]. The pressure channel was selected, the catheter was connected, and the baseline was zeroed. After surgical exposure of the external jugular vein, the distal end was ligated, and a small “V”-shaped venotomy was created. A heparinized catheter was then introduced through the venotomy and advanced sequentially through the superior vena cava into the right ventricle (RV). RVSP was continuously recorded using the BIOPAC system. Following euthanasia, the heart was excised, and the RV, left ventricle (LV), and interventricular septum (S) were separated and weighed. The right ventricular hypertrophy index (RVHI%) was calculated as previously reported.

#### 2.7.4. Histology

Based on our established protocol [19] lung tissue specimens were fixed in 4% paraformaldehyde, dehydrated sequentially through a graded ethanol series, and embedded in paraffin for histological processing. Tissue sections were then stained with hematoxylin and eosin (H&E), and morphological changes were examined under a Leica DMI 3000 B microscope (Leica Microsystems, Wetzlar, Germany).

#### 2.7.5. Immunohistochemistry (IHC)

To evaluate the expression of genes with potential causal relevance to PH, performed immunohistochemistry (IHC) on lung tissues from a 28-day chronic hypoxia mouse model. Lung samples from control (*n* = 3) and HPH mice (*n* = 3) were fixed, paraffin-embedded, and sectioned. IHC staining assessed the expression of α-SMA (1:200, Abcam, Cambridge, UK; Cat# ab7817), FIS1 (1:50, Abiowell, Changsha, China; Cat# AWA11941), OPA1 (1:100, Proteintech, Wuhan, China; Cat# 27733-1-AP), TXNRD1 (1:100, Proteintech, Wuhan, China; Cat# 11117-1-AP), and FUNDC1 (1:200, Abiowell, Changsha, China; Cat# AWA10097). The procedures followed the manufacturers’ instructions. Sections were deparaffinized, rehydrated, and subjected to heat-induced antigen retrieval using citrate buffer at 95 °C for 10 min. Endogenous peroxidase activity was blocked with 0.3% hydrogen peroxide for 10 min. After blocking with 5% bovine serum albumin for 30 min, sections were incubated with primary antibodies overnight at 4 °C. An HRP-conjugated goat anti-rabbit IgG secondary antibody (Gene Tech, Shanghai, China) was applied for 30 min at room temperature. Signal development was performed using a DAB substrate kit, followed by counterstaining of nuclei with hematoxylin. Images were acquired with a digital microscope (Leica, Wetzlar, Germany). Image acquisition and sampling strategy: For each animal, three sections were analyzed, collected at 100 μm intervals to minimize repeated sampling. For each section, three non-overlapping fields were captured using identical acquisition settings, and quantification was averaged per animal prior to group-level statistical analysis.

#### 2.7.6. Transmission Electron Microscopy (TEM)

Distal pulmonary arteries were carefully dissected and isolated from mice under a stereomicroscope. The isolated tissues were immediately immersed in electron microscopy fixative and pre-fixed at room temperature in the dark for 2 h, followed by storage at 4 °C until further processing. Samples were sequentially dehydrated through a graded ethanol series (30%, 50%, 70%, 90%, and 100%), with each step performed for 20 min. Ethanol was then replaced with acetone, and tissues were infiltrated with epoxy resin embedding medium at increasing concentrations and polymerized at 60 °C for 24 h. Embedded samples were cut into ultrathin sections (approximately 50–70 nm) using an ultramicrotome. Sections mounted on copper grids were double-stained with uranyl acetate and lead citrate to enhance contrast. Ultrastructural changes in PASMCs within pulmonary vessels were examined using a transmission electron microscope, with particular attention to mitochondrial morphology, including features consistent with mitochondrial fission/fusion and autophagy-related ultrastructural changes. For each animal, three ultrathin sections were examined, and five non-overlapping fields per section were imaged from comparable vascular regions using identical acquisition settings.

### 2.8. Western Blot Analysis

Proteins were extracted from lung tissue (for the detection of hub MitoDEGs) and mPASMCs (for the detection of hub MitoDEGs, Ferroptosis-related marker proteins after FIS1 transfection) using RIPA buffer(Servicebio, Wuhan, China), which contained 1% PMSF and 1% phosphatase and protease inhibitors. Equal amounts of proteins were then separated by 10% sodium dodecyl sulfate polyacrylamide gels and transferred to polyvinylidene fluoride membranes. The membranes were incubated with primary antibodies for FIS1 (1:1000, Abiowell, Changsha, China), OPA1 (1:1000, Proteintech, Wuhan, China), TXNRD1 (1:1000, Proteintech, Wuhan, China), FUNDC1 (1:1000, Abiowell, Changsha, China), ACSL4 (1:500, ImmunoWay, Plano, TX, USA, Cat# PT0448R). The chemiluminescent signals were detected using a chemiluminescence substrate kit. Densitometry was quantified using the ChemiDoc XRS+ system (Bio-Rad Co., Hercules, CA, USA).

### 2.9. Cell Culture

Mouse PASMCs were obtained from icell (iCell-0110a, iCell Bioscience Inc., Shanghai, China). The cell model was established by culturing the cells in DMEM for 24 h, followed by incubation in a hypoxic environment containing 5% CO_2_, 1% O_2_, and 94% N_2_ to induce hypoxia.

#### 2.9.1. RT-qPCR Experiment

RT–qPCR was performed as follows: Total RNA was extracted using the Tiangen DP419 kitTIANGEN Biotech (Beijing, China), and RNA concentration and purity were assessed by micro-UV spectrophotometry (Allshen, Hangzhou, Ching). Complementary DNA (cDNA) synthesis and quantitative PCR were carried out according to the manufacturer’s instructions. Primer sequences are provided in Table A1 and Table A2, and all primers were synthesized by Shanghai Bioengineering Co., Ltd. (Shanghai, China). Primer specificity was verified by melt-curve analysis showing a single peak, and no-template controls (NTC) were included in each run. Relative gene expression levels were calculated using the 2^−ΔΔCt^ method, with β-ACTIN used as the internal reference. RT–qPCR experiments and reporting followed the MIQE guidelines.

#### 2.9.2. Cell Immunofluorescence

Cell coverslips were sterilized with 70% ethanol and washed three times with PBS before being placed into 24-well plates. mPASMCs were seeded onto coverslips at a density of 1 × 10^4^ cells per well and cultured at 37 °C in a humidified incubator with 5% CO_2_ until reaching 70–80% confluence. Cells were then maintained under normoxia (21% O_2_) or hypoxia (1% O_2_) for 48 h. For immunofluorescence staining, cells were fixed with 4% paraformaldehyde at room temperature for 15 min and washed three times with PBST (5 min each). Cells were permeabilized with 0.1% Triton X-100 for 10 min at room temperature, followed by three PBST washes. After blocking with 5% goat serum for 30 min at room temperature, cells were incubated with the primary antibody (anti-FIS1, 1:100) overnight at 4 °C. Following three PBST washes, cells were incubated with the secondary antibody (goat anti-rabbit IgG, Abbkine, Atlanta, GA, USA; 1:200) for 1 h at room temperature in the dark, and washed three times with PBST. Nuclei were counterstained with DAPI (1 μg/mL) for 10 min at room temperature in the dark, followed by PBST washes. Coverslips were mounted onto glass slides using an anti-fade mounting medium. For each independent experiment, cells were plated in three wells per condition. Three non-overlapping fields per well were imaged using identical acquisition settings, and representative images are shown. Experiments were repeated three times.

#### 2.9.3. Cell Transfection

Cells at 70–80% confluence were seeded into six-well plates and allowed to attach. Cells were then randomly assigned to three groups: control, si-NC, and FIS1 siRNA (siFIS1). Transfection was performed using Lipofectamine 2000 (Invitrogen, Invitrogen, Carlsbad, CA, USA) according to the manufacturer’s instructions. Briefly, siRNA was diluted in Opti-MEM and mixed with diluted Lipofectamine 2000 to allow complex formation at room temperature for 5 min. The complexes were then added to cells to achieve a final siRNA concentration of 25 nM in serum-free medium. After 4–6 h, the medium was replaced with fresh complete medium. At 48 h post-transfection, total RNA and protein were extracted from PASMCs, and transfection efficiency was assessed by RT–qPCR and Western blotting. Three siRNA sequences targeting FIS1 were designed, and the most effective sequence was selected for subsequent experiments. The sequences of nc siRNA, FIS1 siRNA1, and FIS1 siRNA2, as well as the primer sequences for FIS1 and β-ACTIN, are provided in Table A2; all oligonucleotides were synthesized by Shanghai Bioengineering Co., Ltd.

#### 2.9.4. Cell Proliferation Assay Using CCK-8

At 48 h post-transfection, cells were trypsinized and counted to prepare single-cell suspensions. The cells were then cultured under a hypoxic environment (5% CO_2_, 1% O_2_, and 94% N_2_) for 0, and 48 h, with three replicates per time point. At each time point, 10 µL of CCK8 reagent (CK04, DOJINDO, Kumamoto, Japan) was added to each well and incubated for 4 h. The optical density (OD) at 450 nm was measured using a microplate reader(TS 800, BioTek Instruments, Winooski, VT, USA).

#### 2.9.5. mPASMCs Migration Assays

mPASMCs were seeded into 6-well plates and cultured until a uniform confluent monolayer was formed. Cells were serum-starved for 48 h prior to wounding. A straight scratch wound was generated by dragging a sterile 200 µL pipette tip across the cell monolayer in a single, consistent motion. Floating and detached cells were removed by gently washing with PBS. Cells were then maintained in serum-free DMEM and incubated under normoxic or hypoxic conditions for 48 h. Representative images of the same wound regions were captured at 0 h and 48 h using an inverted microscope. Migration was quantified by measuring the wound area using ImageJ 1.54 s, and wound closure was calculated as: wound closure (%) = (A_0_ − A_t_)/A_0_ × 100, where A_0_ is the wound area at 0 h and Aₜ is the wound area at the indicated time point. All experiments were independently repeated three times.

#### 2.9.6. Intracellular ROS Detection

mPASMCs were seeded into 24-well plates and cultured until reaching approximately 70–80% confluency. Following the respective treatments, cells were washed once with phosphate-buffered saline (PBS, pH 7.4), and then incubated with serum-free, phenol red free DMEM containing 10 μM 2′,7′-dichlorodihydrofluorescein diacetate (DCF-DA) at 37 °C for 40 min in the dark. After incubation, cells were washed twice with PBS to remove excess dye. The fluorescence intensity, indicative of intracellular ROS levels, was visualized and captured using a Leica EVOS M7000 fluorescence microscope(Invitrogen, Thermo Fisher Scientific, Waltham, MA, USA). For each independent experiment, cells were plated in three wells per condition. Three non-overlapping fields per well were imaged using identical acquisition settings, and representative images are shown. Experiments were repeated three times.

#### 2.9.7. JC-1 Fluorescence Staining

Paraffin sections were deparaffinized and rehydrated using xylene and graded ethanol, followed by rinsing with water. Cell climbing or frozen sections were directly rinsed with distilled water. The staining area was circled with a PAP pen, and freshly prepared JC-1 working solution was added. Slides were incubated at 37 °C for 20 min in the dark, washed with PBS three times (5 min each), and counterstained with DAPI at room temperature for 10 min in the dark. After PBS washing, slides were mounted with anti-fade mounting medium and imaged using a fluorescence microscope. Total protein content was expressed as nmol/mg protein. For each independent experiment, cells were plated in three wells per condition. Three non-overlapping fields per well were imaged using identical acquisition settings, and representative images are shown. Experiments were repeated three times.

#### 2.9.8. BODIPY Staining

To further assess lipid peroxidation in cells after FIS1 transfection, the lipid peroxidation probe BODIPY 581/591 C11 (Invitrogen) was used. Briefly, 1 mg BODIPY 581/591 C11 was dissolved in 0.1983 mL DMSO to prepare a 10 mM stock solution, and a 10 μM working solution was prepared by diluting 1 μL stock in 999 μL basal culture medium. After removal of the culture medium, cells were washed twice with PBS and then incubated with 1 mL of the 10 μM BODIPY 581/591 C11 working solution at room temperature for 30 min. Cells were subsequently washed three times with serum-free medium to remove extracellular probe, and fluorescence images were acquired immediately using a Motic fluorescence microscope. For each independent experiment, cells were plated in three wells per condition. Three non-overlapping fields per well were imaged using identical acquisition settings, and representative images are shown. Experiments were repeated three times.

#### 2.9.9. Ferrous Iron (Fe^2+^) Measurement

A ferrous iron assay kit was used for determination, and all procedures were performed strictly according to the manufacturer’s instructions. Briefly, mPASMCs were seeded in 6-well plates at a density of 5 × 10^4^ cells per well. After 48 h of transfection, cells were further incubated for 24 h under either normoxic (21% O_2_) or hypoxic (1% O_2_) conditions. Cells were then collected, lysed with the supplied BeyoLysis™ Buffer H for Metabolic Assay, and centrifuged to obtain the supernatant. For Fe^2+^ measurement, Reduction Buffer was added, and samples were mixed with Assay Buffer to a final volume of 190 μL per well, followed by incubation at 37 °C for 30 min in the dark. Subsequently, 10 μL Iron Probe was added and incubated at 37 °C for an additional 30 min in the dark, and absorbance was measured at 593 nm. A standard curve (0.8–50 μM) was generated using the provided standard, and Fe^2+^ concentrations were calculated after blank subtraction. Fe^2+^ content was converted using nmol/well = C (μM) × V (mL) (V = 0.200 mL) and normalized to total protein content determined by a BCA assay.

#### 2.9.10. Statistical Analysis

Statistical analyses were performed using GraphPad Prism (version 10.1.2). The were presented as the mean ± standard error of the mean (SEM), and each experiment was independently repeated at least three times. Differences between two groups were evaluated using an unpaired, two-tailed Student’s *t*-test, whereas comparisons among multiple groups were conducted using one-way analysis of variance (ANOVA). Statistical significance was defined as *p* < 0.05.

## 3. Results

### 3.1. Identification of 69 MitoDEGs in PH

**Figure 1:**
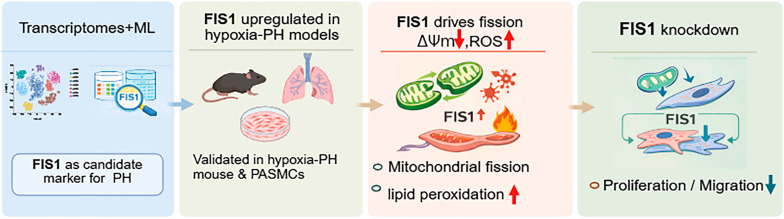
Overview of the study design and analytical workflow. The workflow illustrates the major steps of the study, including integration and batch correction of public transcriptomic datasets, identification of mitochondria-related differentially expressed genes, functional enrichment analyses, candidate gene prioritization, and experimental validation to explore mitochondrial dysfunction and pulmonary vascular remodeling in pulmonary hypertension.

**Figure 2:**
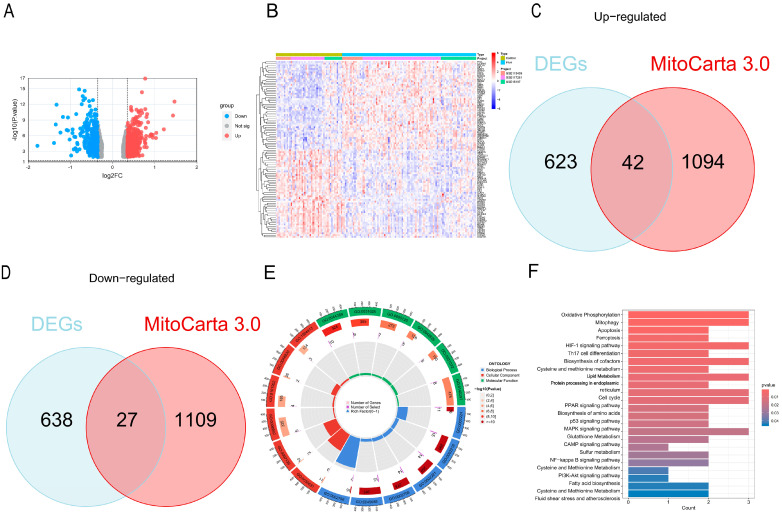
Identification of Differentially Expressed Genes (DEGs). (**A**) Volcano plots depicting DEGs in GSE254617, GSE15197, and GSE117261; (**B**) Heatmaps of DEGs in GSE254617, GSE15197, and GSE117261; (**C**) Venn diagram showing the number of MitoDEGs identified by intersecting upregulated DEGs with mitochondrial-related genes from MitoCarta 3.0; (**D**) Venn diagram showing the number of MitoDEGs identified by intersecting downregulated DEGs with mitochondrial-related genes from MitoCarta 3.0; (**E**) Circular plot of Gene Ontology (GO) enrichment terms for DEGs. Blue denotes biological processes, red indicates cellular components, and green represents molecular functions. Circle colors represent adjusted *p*-values; (**F**) Bubble chart of Kyoto Encyclopedia of Genes and Genomes (KEGG) pathway enrichment, where the *x*-axis shows gene ratios, the *y*-axis shows KEGG terms, and colors indicate adjusted *p*-values.

The overall analytical workflow is summarized in Figure 1. Briefly, three GEO datasets (GSE254617, GSE15197, and GSE117261) were harmonized by batch correction and then subjected to differential expression analysis using DESeq2. Using the thresholds of |log_2_FoldChange| > 0.5 and *p* < 0.05, we identified 665 differentially expressed genes (DEGs) between PH samples and normal controls (NC) (Figure 2A), and the expression patterns of the top DEGs are shown in the heatmap (Figure 2B). Intersecting these DEGs with 1136 mitochondria-related genes from MitoCarta3.0 yielded 69 mitochondrial DEGs (MitoDEGs), including 42 upregulated and 27 downregulated genes (Figure 2C,D). Gene Ontology enrichment indicated that the MitoDEGs were primarily involved in energy metabolism, mitophagy, and membrane transport, with these terms enriched particularly among the upregulated genes (Figure 2E). KEGG pathway analysis further highlighted enrichment in oxidative phosphorylation, metabolic pathways, apoptosis, necroptosis, ribosome-related pathways, and peroxisome signaling (Figure 2F).

### 3.2. Identification of Four Mitochondrial Biomarkers by WGCNA and Machine Learning

**Figure 3:**
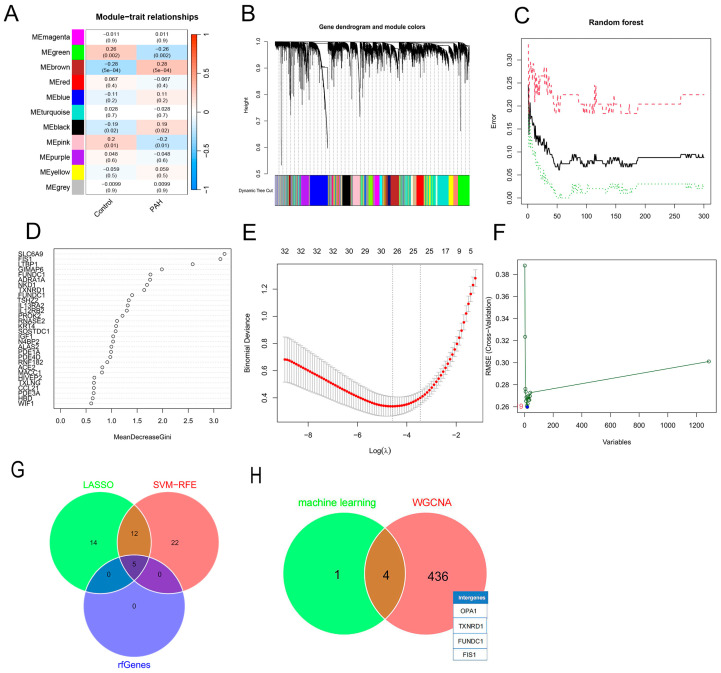
Identification of Potential Core Genes. (**A**) Heatmap depicting the relationship between gene modules and traits based on the weighted gene co-expression network analysis (WGCNA). Darker colors indicate higher correlation, with red representing positive and blue representing negative correlation; (**B**) Dendrogram of gene modules; the upper part shows hierarchical clustering of genes, while the lower part shows clustering of gene modules; (**C**) Relationship between the number of trees and error rate in the random forest (RF) model; (**D**) Gene ranking based on the mean decrease in Gini from the RF model; (**E**) Cross-validation plot of the least absolute shrinkage and selection operator (LASSO) model, where the *x*-axis represents the logarithmic values of the regularization parameter (λ) and the *y*-axis shows the cross-validation error. The vertical dashed line indicates the optimal λ corresponding to the minimum cross-validation error; (**F**) Support vector machine-recursive feature elimination (SVM-RFE) plot illustrating the relationship between the number of features selected and the accuracy/error rate of the model; (**G**) Venn diagram showing the intersection of key genes identified by LASSO, SVM-RFE, and RF; (**H**) Venn diagram showing the intersection of key genes identified by machine learning and WGCNA.

Weighted gene co-expression network analysis (WGCNA) was performed to construct co-expression modules. A total of 21 modules were identified, among which the lightgreen and tan modules showed the strongest negative correlation with the PH phenotype (cor = −0.85, *p* = 1 × 10^−164^; Figure 3A,B). Random forest analysis identified five key genes (Figure 3C,D), LASSO regression identified 26 genes (Figure 3E), and SVM-RFE selected 34 candidate biomarkers (Figure 3F). The intersection of the three algorithms yielded five consensus genes (Figure 3G). After further intersecting with WGCNA modules, four final hub genes were prioritized: FIS1, OPA1, FUNDC1, and TXNRD1 (Figure 3H).

### 3.3. Discriminatory Performance of the Four Hub Genes in Transcriptomic Datasets

**Figure 4:**
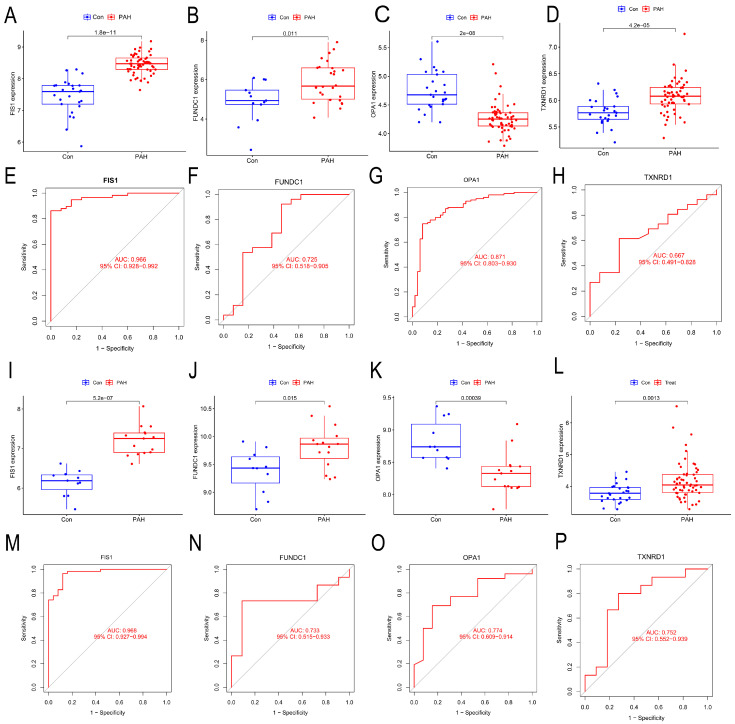
Discriminatory Performance of FIS1, FUNDC1, TXNRD1, and OPA1 in PH. This figure illustrates the expression differences and ROC/AUC-based discriminatory performance of the candidate biomarkers FIS1, FUNDC1, TXNRD1, and OPA1 between pulmonary hypertension (PH) and control samples, shown as boxplots and receiver operating characteristic (ROC) curves. Boxplot (**A**) and ROC curve (**E**) depict FIS1 in the training set, whereas ROC curve (**I**) and boxplot (**M**) show FIS1 in the test set. Boxplot (**B**) and ROC curve (**F**) depict FUNDC1 in the training set, whereas ROC curve (**J**) and boxplot (**N**) show FUNDC1 in the test set. Boxplot (**C**) and ROC curve (**G**) depict OPA1 in the training set, whereas ROC curve (**K**) and boxplot (**O**) show OPA1 in the test set. Boxplot (**D**) and ROC curve (**H**) depict TXNRD1 in the training set, whereas ROC curve (**L**) and boxplot (**P**) show TXNRD1 in the test set.

Receiver operating characteristic (ROC) curve analysis was performed to assess diagnostic performance. FIS1 showed significantly higher expression in PH samples compared to controls, with an AUC of 0.966 in the training datasets and 0.968 in the validation datasets (Figure 4A–D). FUNDC1 yielded AUCs of 0.725 and 0.733, OPA1 yielded AUCs of 0.871 and 0.774, and TXNRD1 had AUCs of 0.667 and 0.782 in the respective (Figure 4E–P). These results highlight FIS1 as the most promising candidate biomarker among the four.

### 3.4. Hypoxia-Induced Pulmonary Hypertension and Mitochondrial Dysfunction in Mice

**Figure 5:**
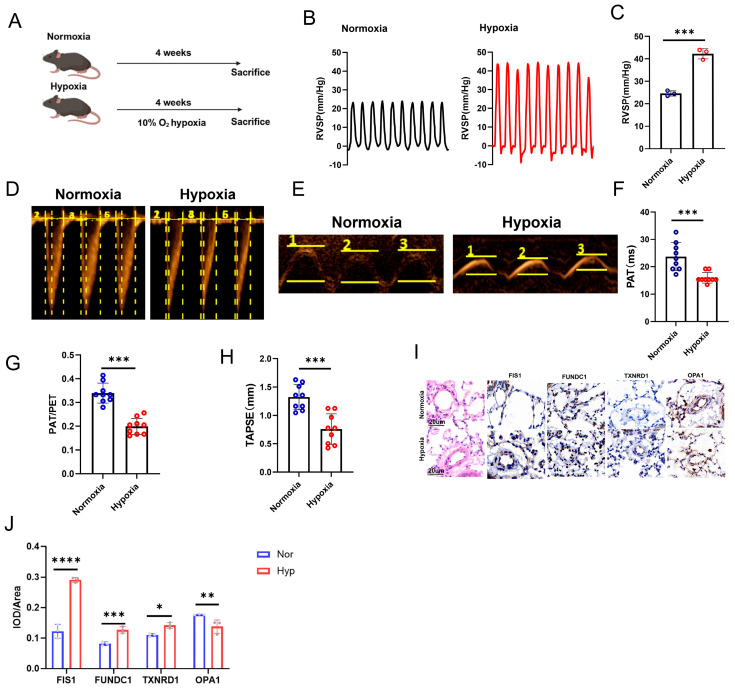
In vivo phenotypic characterization of hypoxia-induced pulmonary hypertension and expression of four hub genes. (**A**) Schematic overview of the hypoxia-induced PH mouse protocol. (**B**) Representative RVSP waveforms. (**C**) Quantification of RVSP. (**D**) Representative pulsed-wave Doppler traces illustrating pulmonary artery acceleration time (PAT) and pulmonary ejection time (PET), and the PAT/PET ratio. (**E**) Representative M-mode images for tricuspid annular plane systolic excursion (TAPSE). (**F**−**H**) Quantification of PAT, PAT/PET, and TAPSE. (**I**) Representative immunohistochemical staining of the four hub genes in lung tissues. (**J**) Quantification of immunohistochemical staining. Data are presented as mean ± SEM; *n* = 3 mice per group. * *p* < 0.05, ** *p* < 0.01, *** *p* < 0.001, **** *p* < 0.0001.

Based on a previously published method [24], HPH was induced in mice using intermittent normobaric hypoxia (Figure 5A). Chronic hypoxia markedly increased right ventricular systolic pressure (RVSP) in the hypoxia group, whereas no significant change was observed in the normoxia (Nor) group, supporting the establishment of an experimental HPH phenotype in mice (Figure 5B,C). To further phenotype hemodynamic changes in this model, transthoracic echocardiography was performed on day 28. Representative echocardiographic images showed that, compared with the Nor group, the Hyp group exhibited significant reductions in pulmonary artery acceleration time (PAT), the PAT/pulmonary ejection time (PET) ratio (Figure 5D–G), and tricuspid annular plane systolic excursion (TAPSE) (Figure 5E,H), reflecting hypoxia-associated hemodynamic impairment accompanied by right ventricular functional decline.

Given that aberrant proliferation and apoptosis resistance of pulmonary vascular cells, together with muscularization of distal pulmonary arterioles, are closely associated with pulmonary vascular remodeling in PH, we further performed histological assessment of distal pulmonary arteries (50–100 μm in diameter). H&E staining revealed pronounced vascular remodeling in response to chronic hypoxia, characterized by marked medial thickening, a representative pathological feature of experimental PH. Collectively, these results support successful phenotypic characterization of the chronic hypoxia induced PH mouse model.

**Figure 6:**
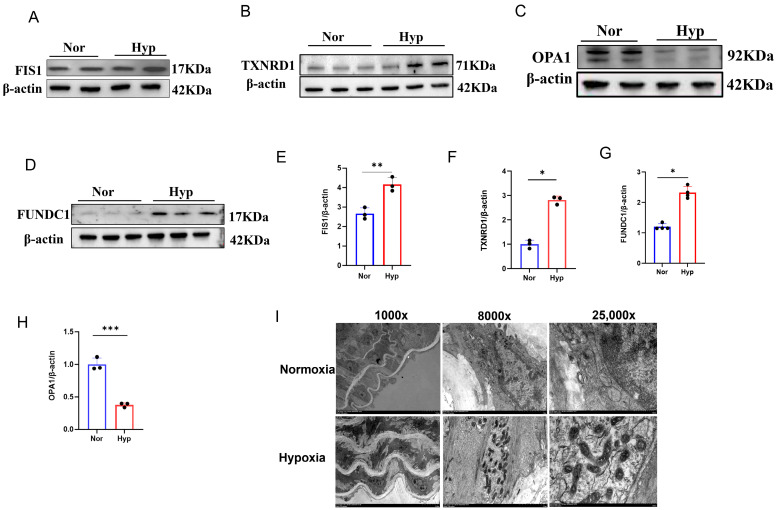
Hypoxia-induced PH phenotyping and mitochondrial fission related alterations in lung tissues. (**A**–**H**) Western blot analysis of FIS1, FUNDC1, OPA1, and TXNRD1 protein levels in lung tissues and corresponding densitometric quantification. Data are presented as mean ± SEM; *n* = 3 mice per group. * *p* < 0.05, ** *p* < 0.01, *** *p* < 0.001. (**I**) Representative transmission electron microscopy (TEM) images of pulmonary artery smooth muscle cells in lung tissues.

Moreover, immunohistochemistry (Figure 5I) and Western blotting (Figure 6A–H) showed that hypoxia exposure decreased OPA1 levels and increased the levels of FIS1, FUNDC1, and TXNRD1 in mouse pulmonary tissues. Transmission electron microscopy (TEM) revealed that PASMCs in PH mice exhibited shortened or fragmented mitochondria, with swelling and cristae disruption, compared with the normoxia group (Figure 6I). Interestingly, mitochondrial fragmentation was accompanied by ultrastructural changes resembling ferroptosis-like features, including condensed mitochondria with increased membrane density, reduced or vanished cristae, and focal outer mitochondrial membrane rupture. These observations suggest that enhanced mitochondrial fission and dysfunction in experimental PH may be linked to ferroptosis-associated stress, a possibility that warrants further investigation.

### 3.5. Immune Cell Infiltration and Its Association with FIS1

To explore whether mitochondrial stress signatures associated with FIS1 coincide with changes in the immune microenvironment in PH, we used ESTIMATE and CIBERSORT to perform immune deconvolution analyses in bulk transcriptomes. PH samples exhibited an altered immune landscape compared with controls, and stratification by FIS1 expression was associated with shifts in several immune cell fractions (Supplementary Figure S1).

### 3.6. Single-Cell Transcriptomics Confirm FIS1 Enrichment in Smooth Muscle Cells

**Figure 7:**
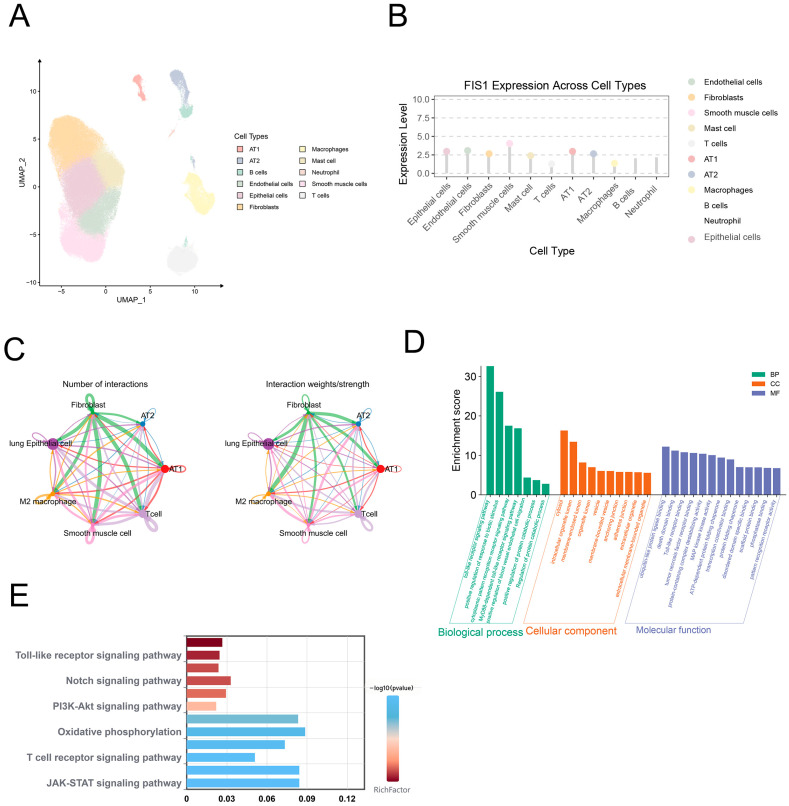
Single-Cell RNA-Seq Analysis of FIS1 Enrichment. (**A**) UMAP plot of 11 distinct cell types in single-cell RNA sequencing data; (**B**) Bar graph showing relative enrichment of FIS1 across different cell types; (**C**) Cell interaction network based on ligand-receptor pairs, with node size representing the cell type’s abundance and line thickness indicating the interaction strength; (**D**) GO enrichment analysis of FIS1-related genes; (**E**) KEGG pathway enrichment analysis of FIS1-related genes.

Single-cell RNA-seq data from GSE144136 were analyzed to determine cell-type specificity. A total of 11 distinct clusters were identified, including AT1, AT2, B cells, endothelial cells, epithelial cells, macrophages, mast cells, neutrophils, smooth muscle cells, T cells, and fibroblasts (Figure 7A). FIS1 expression was broadly distributed but markedly enriched in smooth muscle cells (Figure 7B). Cell communication analysis by CellChat2.0.0 revealed widespread intercellular interactions in PH lungs (Figure 7C), and enrichment analyses further implicated immune pathways (Figure 7D,E).

### 3.7. Expression Patterns of Hub Genes in Hypoxic mPASMCs

**Figure 8:**
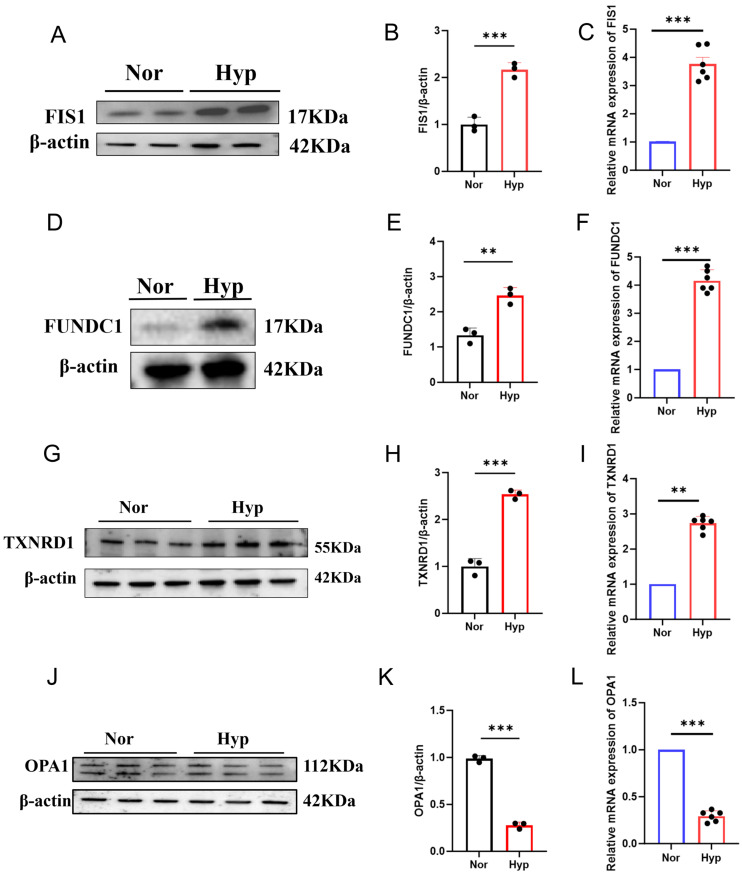
Expression of MitoDEGs in mPASMCs. (**A**–**C**) Western blot and quantitative analysis showing FIS1 expression in mPASMCs; (**D**–**F**) Western blot and quantitative analysis showing FUNDC1 expression in mPASMCs; (**G**–**I**) quantitative analysis showing TXNRD1 expression in mPASMCs; (**J**–**L**) Western blot analysis showing OPA1 expression in mPASMCs. Data are presented as mean ± standard error, *n* = 3. ** *p* < 0.01, *** *p* < 0.001.

To further investigate gene expression in a disease-relevant context, hypoxia-induced mouse PASMCs (mPASMCs) were used. qRT-PCR and Western blot analyses showed upregulation of FIS1, FUNDC1, and TXNRD1 and downregulation of OPA1 under 48-h hypoxia exposure (Figure 8A–L), suggesting involvement in PASMC phenotypic modulation.

### 3.8. Functional Validation of FIS1 in mPASMCs

**Figure 9:**
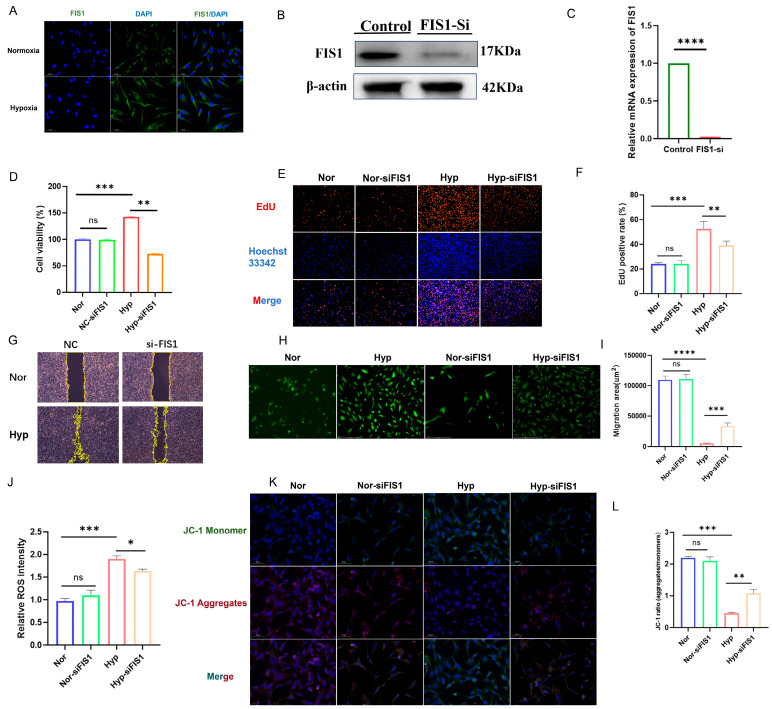
Impact of FIS1 on mPASMCs Proliferation and Migration. (**A**) Immunofluorescence staining of FIS1 expression in mPASMCs; (**B**,**C**) Quantitative real-time PCR (qRT-PCR) and Western blot analysis showing siFIS1 transfection efficiency. Data are presented as mean ± SEM, *n* = 3; (**D**) Cell proliferation assessed by CCK-8 assay, displayed as bar graphs. Data are presented as mean ± SEM, *n* = 3; (**E**) EdU immunofluorescence staining showing the proliferation of mPASMCs in control, hypoxia, and siFIS1 treatment groups. Red fluorescence indicates EdU-positive cells, and DAPI stains cell nuclei (blue); (**F**) EdU-positive cell ratio in mPASMCs among the indicated groups. Data are presented as mean ± SEM, *n* = 3; (**G**) Scratch wound healing assay showing cell migration at 0 h and 48 h in control and siFIS1 groups. Dotted lines indicate the initial wound boundary; (**H**) Quantification of wound healing by measuring the wound area in the indicated groups. Data are presented as mean ± SEM, *n* = 3. (**I**) Fluorescence microscopy images showing ROS levels in mPASMCs from control and siFIS1 groups. Green fluorescence represents DCFH-DA ROS probe signal. (**J**) Relative intracellular ROS intensity in mPASMCs measured by DCFH-DA staining in the indicated groups. Data are presented as mean ± SEM, *n* = 3; (**K**) Fluorescence microscopy images show mitochondrial membrane potential in control and siFIS1-treated mPASMCs. Red fluorescence indicates polarized mitochondria (JC-1 aggregates), while green fluorescence represents depolarized mitochondria (JC-1 monomers). (**L**) Quantification of mitochondrial membrane potential by JC-1 ratio (aggregates/monomers) in control and siFIS1-treated mPASMCs. A higher JC-1 aggregates/monomers ratio indicates more polarized mitochondria, whereas a lower ratio reflects mitochondrial depolarization. Data are presented as mean ± SEM, *n* = 3. * *p* < 0.05, ** *p* < 0.01, *** *p* < 0.001, **** *p* < 0.0001, ns: not significant.

Immunofluorescence staining showed increased FIS1 expression in mPASMCs under hypoxia, as reflected by enhanced fluorescence intensity (Figure 9A). To examine the functional relevance of FIS1, three siRNAs targeting FIS1 were designed, and siFIS1-1 was selected based on knockdown efficiency confirmed by qRT–PCR and Western blotting (Figure 9B,C). CCK-8 and EdU assays showed that FIS1 silencing reduced hypoxia-associated mPASMC proliferation (Figure 9D–F), and wound-healing assays indicated that FIS1 knockdown decreased migratory capacity (Figure 9G,I). In parallel, ROS staining showed that FIS1 depletion attenuated hypoxia-induced intracellular ROS accumulation (Figure 9H,J), consistent with a potential involvement of FIS1 in oxidative stress and mitochondrial injury in this setting.

**Figure 10:**
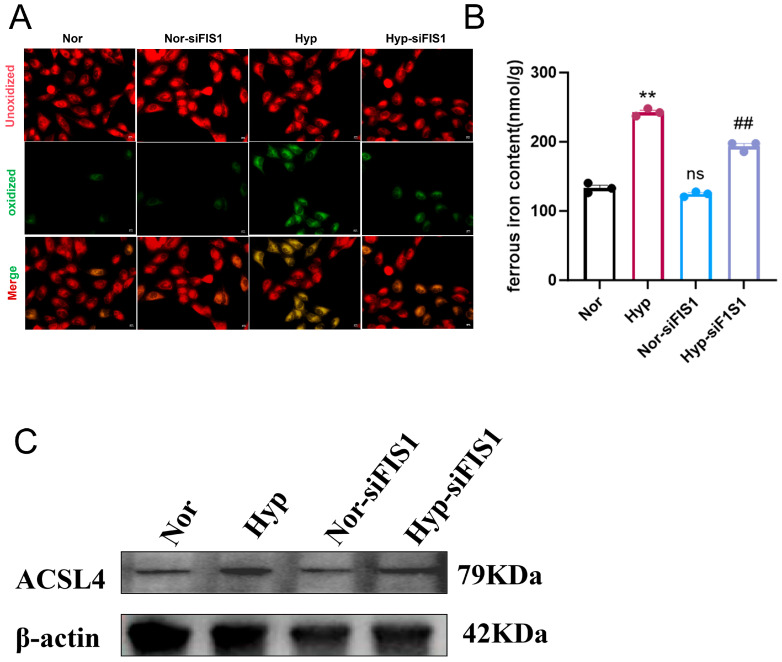
FIS1 knockdown alleviates hypoxia-induced ferroptosis in mPASMCs. (**A**) Representative BODIPY staining images showing lipid peroxidation levels. (**B**) Ferrous iron (Fe^2+^) measurement indicating intracellular Fe^2+^ content. (**C**) Western blot analysis of ACSL4 protein levels. Data are presented as mean ± standard error, *n* = 3. ** *p* < 0.01, ## *p* < 0.01, ns: not significant.

In addition, hypoxia decreased mitochondrial membrane potential (ΔΨm) in mPASMCs, and this reduction was partially alleviated by FIS1 knockdown (Figure 9K,L), suggesting improved mitochondrial function following FIS1 silencing. Given that TEM observations indicated mitochondrial fragmentation accompanied by ferroptosis-like ultrastructural features, we further assessed lipid peroxidation, iron accumulation, and ferroptosis-associated molecular changes. BODIPY-C11 staining revealed increased lipid peroxidation under hypoxia, and this signal was attenuated after FIS1 knockdown (Figure 10A). Consistently, ferrous iron (Fe^2+^) measurements showed that hypoxia increased intracellular Fe^2+^ levels, which were reduced upon FIS1 silencing (Figure 10B). Moreover, the ferroptosis-associated enzyme ACSL4 was upregulated under hypoxia, and its expression was decreased following FIS1 knockdown (Figure 10C). Collectively, these results suggest that FIS1 depletion mitigates hypoxia-associated mitochondrial dysfunction and oxidative stress and is accompanied by reduced lipid peroxidation and iron accumulation, thereby potentially lowering ferroptosis susceptibility in mPASMCs.

## 4. Discussion

PH is a severe pulmonary vascular disorder characterized by sustained pulmonary vasoconstriction and progressive vascular remodeling, which may ultimately progress to right ventricular failure and substantially increase mortality. Although diagnostic and therapeutic strategies for PH have advanced in recent years, and targeted therapies have improved outcomes in a subset of patients with World Health Organization Group 1 pulmonary arterial hypertension (PAH), the overall prognosis remains unsatisfactory. In particular, HPH, which is generally considered within hypoxia-associated Group 3 PH, is driven by chronic exposure to hypobaric hypoxia and exhibits a more complex pathobiological basis; current management remains largely supportive, with a lack of disease-specific interventions supported by robust evidence [25,26]. Therefore, delineating key pathogenic processes in HPH and identifying translatable molecular targets are of substantial clinical significance.

Mitochondria serve as a central hub for cellular energy metabolism and signal transduction, and regulate ATP production, redox homeostasis, cell-fate decisions, and immune–inflammatory signaling [27]. Under chronic hypoxic conditions, mitochondria are highly sensitive to fluctuations in oxygen availability, and hypoxia-driven remodeling of mitochondrial morphology and function has been closely linked to pulmonary vascular cell phenotypic switching and vascular remodeling [28]. Accumulating evidence suggests that disrupted mitochondrial dynamics and coupled mitochondrial quality-control processes can promote abnormal proliferation, migration, and apoptosis resistance of PASMCs, thereby contributing to medial thickening and luminal narrowing [29]; however, the key molecular nodes underlying HPH-associated mitochondrial dynamics dysregulation, as well as their value for molecular stratification and therapeutic intervention, require more systematic validation.

Through the integration of weighted gene co-expression network analysis with three machine learning algorithms (LASSO, SVM-RFE, and random forest), the study identified FIS1, FUNDC1, TXNRD1, and OPA1 as key candidate biomarkers. Among these, FIS1 demonstrated the highest discriminatory performance, with area under the curve (AUC) values of 0.966 and 0.968 in the training and validation datasets, respectively. This multi-level analytical approach provides a comprehensive framework for identifying clinically relevant biomarkers in complex diseases such as PH. Beyond transcriptomic feature selection, AI and machine learning approaches in PH are increasingly used to integrate multimodal information, including clinical variables, echocardiography, hemodynamic measures, imaging features, and multi-omics profiles, to support refined phenotyping and risk stratification. This broader direction highlights the potential value of combining molecular signals with clinical and imaging data, while also requiring rigorous external validation and careful attention to heterogeneity and interpretability. In this context, our study focuses on transcriptome based prioritization of mitochondria related candidates and provides a foundation for future multimodal extensions [30].

Functionally, FUNDC1 acts as a mitophagy receptor on the outer mitochondrial membrane. Under hypoxic stress, it facilitates the selective removal of damaged mitochondria. While moderate activation maintains mitochondrial quality, excessive FUNDC1 expression can stabilize HIF-1α, leading to enhanced proliferation and apoptosis resistance in pulmonary artery smooth muscle cells, thereby promoting vascular remodeling [31,32]. TXNRD1, which encodes thioredoxin reductase 1, plays a critical role in cellular redox homeostasis. Its downregulation under hypoxic conditions results in elevated reactive oxygen species, endothelial dysfunction, and aberrant smooth muscle proliferation [33,34]. OPA1, located in the inner mitochondrial membrane, is essential for maintaining cristae integrity and oxidative phosphorylation. Reduced OPA1 expression impairs mitochondrial structure and bioenergetic capacity, contributing to right ventricular dysfunction [35]. FIS1 is anchored to the mitochondrial outer membrane and promotes mitochondrial fission. It exhibits upregulation in PH and high enrichment in pulmonary artery smooth muscle cells. Experimental validation demonstrated that FIS1 enhances cellular proliferation and ROS accumulation in these cells, thereby driving pulmonary vascular remodeling and establishing its central role in PH pathogenesis.

Mitochondria integrate cellular metabolism and stress responses and have been implicated in immune and inflammatory signaling, potentially linking metabolic remodeling to inflammatory changes in PH [36,37,38]. In our exploratory immune deconvolution analysis, FIS1 expression was associated with variation in the estimated abundance of several immune cell subsets, showing positive associations with resting CD4^+^ memory T cells and activated dendritic cells and negative correlations with follicular helper T cells and M1 macrophages [39,40,41,42]. Importantly, these results are computational and correlative and may be influenced by differences in tissue cellular composition, cell-type proportions, or secondary inflammatory remodeling, rather than indicating a direct immunoregulatory role of FIS1. Therefore, these observations should be interpreted as generating testable hypotheses, and future studies with dedicated immune-focused experiments will be required to determine whether FIS1 is causally linked to immune alterations in PH. Mitochondrial fragmentation and mitochondrial stress signaling, including mitoROS-related pathways, may also coincide with inflammatory activation in PH and warrant further investigation [43,44].

Single-cell RNA sequencing confirmed the cell-type specificity of FIS1, predominantly expressed in smooth muscle cells. Structural studies have shown that FIS1 is a transmembrane protein with an N-terminal HIP-TPR-like domain that recruits fission regulators, a flexible linker that undergoes SUMOylation to modulate activity, and a C-terminal membrane-anchoring region. Pharmacological inhibition of mitochondrial fission complexes represents a potential therapeutic approach in PH. Small-molecule inhibitors including Mdivi-1, P110, and Drpitor1a have demonstrated efficacy in preclinical models by reducing vascular remodeling and improving hemodynamic outcomes. In alignment with these findings, in vitro experiments revealed that silencing FIS1 reduced PASMC proliferation, migration, and ROS production, underscoring its therapeutic relevance [14,45].

Among the candidate genes identified in this study, FIS1 showed strong ROC/AUC-based discriminatory performance in both the training and validation datasets, suggesting that it may be associated with mitochondrial dynamics abnormalities in hypoxia-related PH. It should be noted that prior studies linking FIS1 to hypoxic PH remain relatively limited and have largely focused on how post-translational regulation of FIS1 in pulmonary arterial endothelial cells influences mitochondrial remodeling and disease phenotypes [46]. In contrast, whether FIS1, as an outer mitochondrial membrane fission-associated protein, directly drives excessive mitochondrial fission in PASMCs through its intrinsic fission-regulatory function and subsequently modulates downstream events such as lipid peroxidation and iron homeostasis disruption still lacks direct and systematic mechanistic evidence.

Mechanistically, FIS1 is anchored to the outer mitochondrial membrane, participates in the organization and regulation of the fission apparatus, and is functionally linked to DRP1-dependent membrane scission events [47,48]. Meanwhile, accumulating evidence supports a functional coupling between mitochondrial dynamics and ferroptosis susceptibility: mitochondrial fragmentation accompanied by DRP1 activation is observed during ferroptosis, and interventions targeting key nodes of mitochondrial dynamics, such as inhibiting DRP1 or enhancing mitochondrial fusion programs, can attenuate ferroptotic cell death.

In this context, our in vivo and in vitro results support the following working model: under hypoxic stress, FIS1 upregulation is accompanied by enhanced mitochondrial fission in PASMCs, together with a decline in mitochondrial membrane potential and increased ROS accumulation. These mitochondrial changes are associated with ferroptosis-associated alterations, including increased lipid peroxidation and disrupted iron homeostasis, and may coincide with enhanced ferroptosis susceptibility as well as PASMC hyperproliferation and migration during pulmonary vascular remodeling [48,49]. Importantly, FIS1 knockdown partially mitigated mitochondrial injury and reduced ferroptosis-associated changes, while attenuating PASMC proliferation and migration. Together, these findings support a potential link between mitochondrial dynamics and ferroptosis-associated stress in hypoxia-related PH and suggest that targeting FIS1 may have mechanistically informed therapeutic relevance.

It should be noted that, although this study integrated multi-omics analyses with in vivo and in vitro experiments to characterize mitochondrial dynamics imbalance in pulmonary hypertension and to prioritize FIS1, FUNDC1, TXNRD1, and OPA1, several limitations remain. Some in vivo validations used relatively small sample sizes, which may limit statistical power and increase uncertainty in effect size estimates; therefore, these results should be interpreted cautiously and validated with expanded sample sizes in future studies. In addition, the causal relationships linking mitochondrial dynamics dysregulation to ferroptosis are not fully defined. Future work should determine whether FIS1-driven mitochondrial fission acts upstream of ferroptosis susceptibility and delineate the key pathways involved in lipid peroxidation and iron homeostasis. Moreover, further validation in systems that more closely reflect human disease, including primary human pulmonary vascular cells, cell–cell interaction models, and cell type-specific animal models, will be important to clarify cell type specific actions and strengthen mechanistic rigor and translational relevance.

Overall, based on transcriptomic datasets derived from PH models that include the HPH subtype, combined with multi-algorithm machine learning selection and in vivo and in vitro experimental validation, this study systematically characterized the molecular landscape of mitochondrial homeostasis disruption in hypoxia-related PH and identified FIS1, FUNDC1, TXNRD1, and OPA1 as key candidate genes. Our findings further suggest that mitochondrial dynamics dysregulation is associated not only with mitochondrial functional impairment and enhanced oxidative stress, but may also increase cellular susceptibility to ferroptosis by promoting lipid peroxidation and disrupting iron homeostasis, thereby contributing to the maintenance of abnormal PASMC phenotypes and pulmonary vascular remodeling. Among these candidates, FIS1 exhibited consistent ROC/AUC-based discriminatory performance in the analyzed datasets, and its upregulation co-occurred with excessive mitochondrial fission, loss of mitochondrial membrane potential, ROS accumulation, and ferroptosis-related phenotypic alterations; conversely, FIS1 knockdown partially alleviated mitochondrial injury, reduced ferroptosis-related stress, and suppressed PASMC proliferation and migration. Collectively, these results indicate that mitochondrial functional impairment secondary to enhanced mitochondrial fission in hypoxia-related HPH may coincide with ferroptosis-associated processes, including lipid peroxidation and iron dyshomeostasis, thereby increasing ferroptosis susceptibility and contributing to pulmonary vascular remodeling. This work provides new molecular insights into HPH by highlighting coordinated regulation between mitochondrial dynamics and ferroptosis, which may aid future therapeutic exploration.

## 5. Conclusions

In summary, this study further supports the value of FIS1 as a potential candidate biomarker and candidate target for future evaluation in HPH. To substantiate this conclusion, we integrated transcriptomic datasets derived from PH models that include the HPH subtype and performed systematic validation using both in vivo models and in vitro models. Through a combined bioinformatics and experimental strategy, we delineated the key molecular features of mitochondrial homeostasis disruption in HPH and identified FIS1, FUNDC1, TXNRD1, and OPA1 as core candidate genes. Our results showed that hypoxia-induced upregulation of FIS1 was accompanied by excessive mitochondrial fission, loss of mitochondrial membrane potential, and ROS accumulation, together with ferroptosis-associated phenotypic alterations such as enhanced lipid peroxidation and iron dyshomeostasis. Moreover, FIS1 knockdown partially alleviated mitochondrial injury, reduced ferroptosis-related stress, and suppressed PASMC hyperproliferation and migration, thereby attenuating pulmonary vascular remodeling. Collectively, these findings provide mechanistic insight into the interplay between mitochondrial dynamics and ferroptosis susceptibility in HPH and motivate further studies on molecular stratification and therapeutic exploration.

## Data Availability

The datasets used and analyzed in this study are available in GEO under accession numbers GSE254617, GSE15197, and GSE117261.

## Supplementary Materials

The following supporting information can be downloaded at: https://www.mdpi.com/article/10.3390/cells15030301/s1, Figure S1. Correlation of FIS1 Expression with Immune Infiltration. (**A**,**B**) Violin plots showing the expression levels (**A**) and relative proportions (**B**) of immune cell types in normal and PH lung tissues; (**C**) Heatmap showing the abundance of 28 immune cell subtypes in samples; (**D**) ESTIMATE algorithm-based immune infiltration evaluation comparing high and low FIS1 expression groups; (**E**) CIBERSORT deconvolution algorithm-based immune cell infiltration analysis; (**F**) Scatter plot showing the correlation between FIS1 expression and the infiltration levels of various immune cell types. Statistical significance in (**B**,**D**) assessed by Wilcoxon rank-sum test (* *p* < 0.05, ** *p* < 0.01, *** *p* < 0.001).

## Abbreviations

The following abbreviations are used in this manuscript:

PH	Pulmonary hypertension
HPH	Hypoxic pulmonary hypertension
PVR	Pulmonary vascular remodeling
PASMCs	Pulmonary artery smooth muscle cells
mPASMCs	Mouse pulmonary artery smooth muscle cells
DEGs	Differentially expressed genes
MitoDEGs	Mitochondria-related differentially expressed genes
WGCNA	Weighted gene co-expression network analysis
SVM-RFE	SVM-recursive feature elimination
LASSO	Least absolute shrinkage and selection operator
ROC	Receiver operating characteristic
AUC	Area under the curve
RVSP	Right ventricular systolic pressure
PAT	Pulmonary artery acceleration time
PET	Pulmonary artery ejection time
TAPSE	Tricuspid annular plane systolic excursion
TEM	Transmission electron microscopy
IHC	Immunohistochemistry
ROS	Reactive oxygen species
GO	Gene Ontology
KEGG	Kyoto Encyclopedia of Genes and Genomes
GEO	Gene Expression Omnibus

## Appendix A

**Table A1:** Sequence information.

Name	Sequence (5′-3′)
FUNDC1	AGCGATGACGAATCATACGAAG
	CCACCCATTACAATCTGAGTAGC
TXNRD1	GGGTCCTATGACTTCGACCTG
	AGTCGGTGTGACAAAATCCAAG
OPA1	TGGAAAATGGTTCGAGAGTCAG
	CATTCCGTCTCTAGGTTAAAGCG
FIS1	TGTCCAAGAGCACGCAATTTG
	CCTCGCACATACTTTAGAGCCTT
β-actin	TCCCTGGAGAAGAGCTATGAG
	TGGCATAGAGGTCTTTACGGA

**Table A2:** Sequence information.

Name	Sequence (5′-3′)
NC siRNA	UUCUCGACGUGUCACGUTT
	ACGUGACACGUUCGGAGAATT
FIS1 siRNA-1	GGUUCGAAGCAAAUACAAU/dT//dT/
	AUUGUAUUUGCUUCGAACC/dT//dT/
FIS1 siRNA-2	GCUGGUUCUGUGUCCAAGA/dT//dT/
	UCUUGGACACAGAACCAGC/dT//dT/
FIS1 siRNA-3	GGCUCUAAAGUAUGUGCGA/dT//dT/
	UCGCACAUACUUUAGAGCC/dT//dT/
FIS1	TGTCCAAGAGCACGCAATTTG
	CCTCGCACATACTTTAGAGCCTT
β-actin	TCCCTGGAGAAGAGCTATGAG
	TGGCATAGAGGTCTTTACGGA
